# Spontaneous heterotopic pregnancy with tubal rupture: a case report and review of the literature

**DOI:** 10.4076/1752-1947-3-8153

**Published:** 2009-06-10

**Authors:** Rimpy Tandon, Poonam Goel, Pradip Kumar Saha, Lajya Devi

**Affiliations:** 1 Department of Obstetrics & Gynecology and Pathology, Government Medical College & Hospital, Sector 32-B, Chandigarh-160 030, India

## Abstract

**Introduction:**

Heterotopic pregnancy is diagnosed as the presence of two gestations simultaneously. This is a rare situation with a reported prevalence of 0.08% in normal conception.

**Case presentation:**

We report a case of a 24-year-old primigravida of Indian origin who was seen in the emergency department with a diagnosis of a ruptured ectopic pregnancy. A careful ultrasound assessment led to the diagnosis of a heterotopic pregnancy. Immediate surgical intervention with supportive measures resulted in a successful outcome.

**Conclusion:**

An obstetrician should keep in mind the occurrence of a heterotopic pregnancy while dealing with pregnant females. The ectopic gestation invariably ruptures over a period of time leaving the patient in an emergency situation. A quick assessment and careful handling of the normal gestation can lead the patient to term with gratifying results.

## Introduction

Heterotopic pregnancy (HP) is diagnosed in the presence of simultaneous gestations at two or more implantation sites. It was first reported in the year 1708 as an autopsy finding [1]. Its occurrence is rare in spontaneous conception with an incidence of 1:30,000 [1], while in assisted reproductive techniques (ART), the incidence is found to be as high as 1% [1]. We report a case of HP in a natural conception cycle that presented with tubal rupture.

## Case presentation

A 24-year-old primigravid woman of Indian origin and married for one year was seen in our emergency department with a history of a brief episode of loss of consciousness and acute pain in her abdomen of four hours duration. She was 8 weeks pregnant. It was a spontaneous conception and there was no past history of abortion, infertility, pelvic inflammatory disease or any history of abdominal surgery. On examination, she was pale with a pulse rate of 120 per minute and blood pressure of 80/60 mmHg. Abdominal examination revealed diffuse, lower abdominal tenderness with significant guarding and rigidity. Pelvic examination revealed an anteverted, enlarged, soft and tender uterus corresponding to 8 weeks of pregnancy. In addition, a tender mass was also palpable in her right adnexa. Cervical movements were painful but there was no bleeding. After initial resuscitation with intravenous fluids, she was further investigated. Her hemoglobin was 4.2 gm/dl with a normal white blood count (WBC) and platelet count. Urine for HCG (human chorionic gonadotropin) was positive. Transvaginal sonography revealed an 8-week intrauterine viable pregnancy and a 3.3 × 2.2 cm echogenic mass near her right ovary with a 1.6 × 1.0 cm central anechoic area. A moderate amount of fluid was present in the cul-de-sac and in Morrison's space and a diagnosis of heterotopic pregnancy with tubal rupture was made. An emergency laparotomy on the patient revealed an 8-week gravid uterus and rupture of the right tube near the fimbrial end and the presence of approximately 1.5 liters of hemoperitoneum. Right salpingectomy with removal of the hemoperitoneum and peritoneal lavage was performed. She was transfused with four units of blood during and after the surgery and her postoperative period was uneventful. Histopathology of the resected specimen showed the presence of chorionic villi confirming a viable pregnancy. The patient was discharged and followed-up regularly in the antenatal clinic. At 38 weeks gestation she went into spontaneous labor and delivered a healthy male baby weighing 2.6 kg with no congenital malformation. Postnatal recovery was uneventful and both mother and baby were discharged on the third postpartum day.

## Discussion

HP is diagnosed in the presence of multiple pregnancies with one or more intrauterine pregnancies co-existing with an ectopic pregnancy. The ectopic pregnancy can be tubal, ovarian, cervical, cornual or abdominal. Tubal ectopic pregnancies are the most common. The occurrence of a heterotopic pregnancy is considered rare in natural conception cycles with an incidence of 0.08%, but incidence increases to as high as 1% with assisted reproductive techniques [1]. This is because of transfer of embryos by ART techniques into affected tubes and peristaltic movements do not expel these embryos. The common factors that predispose to occurrence of ectopic pregnancy are tubal surgery and pelvic inflammatory diseases [1,3].

Early diagnosis of HP is often difficult because of the absence of clinical symptoms. Reece et al [1] defined abdominal pain, adnexal mass, peritoneal irritation and an enlarged uterus as signs and symptoms suspicious of HP. Transvaginal ultrasound and assessment of the whole pelvis, even in the presence of intrauterine pregnancy, can be an important aid in the diagnosis of HP [1]. Further, visualization of heart activity in both intrauterine and extrauterine gestation confirms the diagnosis of HP [1].

The advent of ultrasound (USG) has not changed diagnostic ability over a period of time. In a review of the literature of all cases of HP from 1971 to 1993, out of 112 cases, 46 were diagnosed by USG while 66 were diagnosed at laparoscopy or laparotomy. The recent literature review from 1994 to 2004 also showed that out of 80 cases, 21 were diagnosed by USG and 59 at laparoscopy or laparotomy [1]. One of the reasons for this unexpected observation is that HP is a rare condition and most patients with HP present in the emergency department with symptoms of a rupture of ectopic component. Thus, a preoperative diagnosis of HP is still a challenge.

Serial β-HCG levels are not of much significance in the diagnosis of HP as subnormal hormone production by an ectopic pregnancy may be masked by the higher placental production from the intrauterine pregnancy. Culdocentesis is an important aid in diagnosis when hemoperitoneum is present [1] as echogenic pelvic fluid is more important than anechoic fluid because it indicates the presence of peritoneal hemorrhage.

The standard treatment for ectopic pregnancy is surgery by laparoscopy or laparotomy depending upon the condition of the patient. The main aim of the surgery should be the preservation of the intrauterine pregnancy with minimal manipulation of the uterus. Medical treatment for ectopic pregnancy with an intact tube is a local injection of potassium chloride. Fertility results have been found to be the same after laparoscopy or laparotomy. Conservative or radical surgery may be done depending upon the condition of the contralateral tube; a review by Clausen I [1] demonstrated no difference in rates of IUP after conservative or radical surgery. The prognosis of IUP is favorable in 60 to 70% of cases; Smith and Siddique reported a survival rate between 35% and 54% in 1971 [1]. In the study by Tal et al [1] 66% proceeded to full term while in the study by Barrenetxea et al, 69% proceeded to full term [1]. The improvement in IUP survival rate is probably due to better diagnostic and treatment developments and close follow-up of patients after ART techniques.

## Conclusion

Heterotopic pregnancy can occur in the absence of any predisposing risk factors, and the detection of an intrauterine pregnancy does not exclude the possibility of the simultaneous existence of an ectopic pregnancy. Hence, in all patients of reproductive age, even in the presence of an intrauterine pregnancy, a complete review of the whole pelvis including adnexa should be done at the time of ultrasound to rule out the presence of a heterotopic pregnancy.

## Abbreviations

ART: assisted reproductive techniques; HP: heterotopic pregnancy; IUP: intrauterine pregnancy; USG: ultrasound.

## Consent

Written informed consent was obtained from the patient for publication of this case report. A copy of the written consent is available for review by the Editor-in-Chief of this journal.

## Competing interests

The author(s) declare that they have no competing interests.

## Authors' contributions

RT was involved in the patient management and wrote the paper. PG and PKS were involved in the patient management. LD was involved in the follow-up of the patient. All authors read and approved the final manuscript.
